# Anxiolytic-like Activity, Antioxidant Properties, and Facilitatory Effects on the Short-Term Memory Retention of Molsidomine in Rats

**DOI:** 10.3390/life14030306

**Published:** 2024-02-26

**Authors:** Liliana Mititelu-Tartau, Maria Bogdan, Liliana Lăcrămioara Pavel, Ciprian Rezus, Cezar Ilie Foia, Nicoleta Dima, Irina Luciana Gurzu, Ana-Maria Pelin, Beatrice Rozalina Buca

**Affiliations:** 1 Department of Pharmacology, Faculty of Medicine, ‘Grigore T. Popa’ University of Medicine and Pharmacy, 700115 Iasi, Romania; lylytartau@yahoo.com (L.M.-T.); cezar030699@gmail.com (C.I.F.); beatrice-rozalina.buca@umfiasi.ro (B.R.B.); 2 Department of Pharmacology, Faculty of Pharmacy, University of Medicine and Pharmacy, 200349 Craiova, Romania; 3 Department of Morphological and Functional Sciences, Faculty of Medicine and Pharmacy, ‘Dunarea de Jos’ University, 800010 Galati, Romania; 4 Internal Medicine Department, 3rd Medical Clinic, ‘Sf. Spiridon’ Clinical Emergency Hospital, ‘Grigore T. Popa’ University of Medicine and Pharmacy, 700115 Iasi, Romania; ciprianrezus@yahoo.com (C.R.); nicoleta2006r@yahoo.com (N.D.); 5 Department of Preventive Medicine and Interdisciplinarity, Faculty of Medicine, ‘Grigore T. Popa’ University of Medicine and Pharmacy, 700115 Iasi, Romania; irina-luciana.gurzu@umfiasi.ro; 6 Department of Pharmaceutical Sciences, Faculty of Medicine and Pharmacy, ‘Dunarea de Jos’ University, 800010 Galati, Romania; anapelin@gmail.com

**Keywords:** molsidomine, V-pyrro/NO, actimeter, Y-maze, rats

## Abstract

Compelling evidence indicates that nitric oxide (NO) exerts a significant influence on the central nervous system, participates in the modulation of neurotransmitter release, contributes to the regulation of cognitive functions, and plays a crucial role in modulating various aspects of neural activity. We aimed to explore the influence of two NO donors, molsidomine (MSD) and V-pyrro/NO, on the innate spontaneous psychomotor abilities and short-term memory in rats. Using an actimeter test, the locomotor activity, stress-sensitive behavior, and anxiety level were investigated. The influence on the animal`s cognitive functions was evaluated usingthe Y-maze test to assess the spontaneous alternation percentage, number of arms visited, number of alternations, and the preference index. Four distinct groups of five white male Wistar rats were exposed to the intraperitoneal treatments as follows: Control batch—0.3 mL/100 g of body weight saline solution, Mg batch—200 mg/kbwof magnesium chloride, MSD batch—1 mg/kbw of molsidomine, and V-pyrro/NO batch—5 mg/kbwof V-pyrro/NO. The intraperitoneal administration of MSD resulted in a significant reduction in spontaneous behavior and exploratory skills but was less pronounced than the positive control drug, magnesium chloride. Conversely, treatment with V-pyrro/NO led to only a slight decrease in horizontal movements during the actimeter test. MSD administration, but not V-pyrro/NO, notably increased the rate of spontaneous alternation in the Y-maze test. Additionally, the use of MSD resulted in an increase in the blood level of brain-derived neurotrophic factor and the intensification of the antioxidant enzymes, superoxide dismutase, and glutathione peroxidase activity. In our experimental setup, we demonstrated that MSD exposure led to a decrease in spontaneous behavior, showed anxiolytic effects and antioxidant activity, and improved spatial memory acquisition in rats.

## 1. Introduction

Originally known as “endothelium-derived relaxing factor”, nitric oxide (NO) has been thoroughly researched in recent years, being a messenger with a key role in the body, both in the central nervous system (CNS) and the peripheral one. It serves as a messenger within and outside cells, facilitating various signaling pathways in target cells [1]. This molecule is recognized for its significant involvement in numerous physiological processes, such as neuronal signaling, regulation of ion channels, cardiovascular function, inflammatory responses, and immune defense capabilities [2,3,4,5,6]. It was also proposed that NO has functions in overseeing cell bioenergetics, managing cell demise, and governing the balance between oxygen supply and demand for the maintenance of cellular integrity [1,7,8,9].

Harnessing the impressive therapeutic potential of NO remains an ongoing challenge even today. Despite the obvious potential of NO, no remarkable progress has been made in obtaining NO donors, given the scientific excitement generated by the identification of this molecule as an important biological messenger [10].

The outline of the role of NO as a free radical in the CNS created a new concept in terms of neuronal communication, thus overcoming the classical patterns of information transmission based on chemical mediators, where the information is transmitted unidirectionally through synapses [1,11,12,13].

A particular characteristic of NO, which distinguishes it from other neurotransmitters in the CNS, is its ability to diffuse particularly rapidly. This property manifests itself both in water and in the lipid environment, thus allowing a three-dimensional spread of the carried message, independent of the existence of membranes [14,15].

Even though it is very simple in structure, NO has multiple chemical properties and varied biological actions. It is highly reactive, even though its half-life is only a few seconds [1,16].

NO has been shown to play an important role in the signaling and regulation of glutamatergic synapses by modulating ion channel firing, membrane fusion, fission, and compartmentalization or by facilitating protease-mediated protein degradation [17]. In particular, S-nitrosylation has been suggested to be involved in the pathogenesis of various neurodegenerative disorders, such as Parkinson’s disease, amyotrophic lateral sclerosis, multiple sclerosis, and Alzheimer’s disease [11,18,19]. Neuronal inflammation, which characterizes these pathological states, is largely associated with pathogenic pathways involved in NO production [20]. If the physiological control of these signaling pathways fails, the pathological effects of NO lead to neuroinflammatory and neurodegenerative processes [21,22,23].

However, a multitude of studies have illustrated that the signaling of NO functions as a switch, shifting between the detrimental and restorative stages of neuronal remodeling. Additionally, each NO donor displays distinct physical and chemical characteristics, leading to diverse physiological consequences [24].

The therapeutic potential of NO faces significant challenges due to its short biological lifespan, instability during storage, and potential toxicity [25]. A promising approach to overcome these limitations and increase the amount of NO released involves the development of NO-donor compounds. Over the past two decades, extensive research efforts have been dedicated to developing the most efficient materials for generating and releasing NO in clinical therapies [26,27,28]. Numerous compounds, such as N-diazeniumdiolates, nitrosothiols, nitrosohydroxylamines, and nitrosyl metal complexes, have been engineered to chemically stabilize and release NO in a controlled manner. These compounds have found applications in various biomedical fields [26,29,30].

Molsidomine (MSD) is employed in treating various forms of coronary diseases as it functions as a NO-releasing agent. It exerts its influence through NO and achieves enhanced myocardial perfusion by dilating the coronary arterial system [31]. Additionally, it increases the cardiac preload and elevates the peripheral venous capacitance, thus diminishing the oxygen demand [32]. Preliminary research conducted previously demonstrated that molsidomine successfully alleviated non-spatial recognition memory deficits, stereotypies, and ataxia induced by the NMDA receptor antagonist MK-801 in rats, but the mechanisms involved have not been completely deciphered [33,34].

Pitsikas et al. showcased that a dosage of 4 mg of molsidomine, as opposed to 2 mg, resulted in significant impacts on spatial memory in healthy rats without motor impairment, thereby reducing the typical decline in natural forgetting related to object-recognition memory [35].

The same authors contended that while 1 mg/kg of MSD on its own does not enhance performance deficits in object recognition, beneficial impacts on cognitive functions arise when it is combined with the non-competitive N-methyl-D-aspartate (NMDA) receptor antagonist memantine (3 mg/kg) within the same rat experimental model [36].

In another study, Pitsikas et al. revealed anxiolytic-like effects of 2 mg/kg of molsidomine (but not of 1 mg/kg or 4 mg/kg) in the light/dark box and open field tests in rats, effects that are not linked to modifications in locomotor activity [37].

The O^2^-vinyl 1-(pyrrolidin-1-yl)diazen-1-ium-1,2-diolate compound V-pyrro/NO is a NO donor in different tissues in the body, especially in the liver. It is known for its favorable effects, improving splanchnic circulation and recovering liver fibrotic damage in various pathological states, and has proven to have protective effects on acetaminophen-induced kidney damage [38,39,40].

In this experimental research, we aimed to investigate the influence of two NO donors, molsidomine and V-pyrro/NO, on spontaneous psychomotor abilities and cognitive functions in rats. Our focus was on assessing the influence of this drug on innate memory rather than on chemically induced cognitive deficits.

## 2. Materials and Methods

### 2.1. Chemicals

MSD (catalogue code: Y0000821, molecular weight: 242.23) and magnesium chloride (anhydrous ≥ 98%, catalogue code: M8266, molecular weight: 95.21) were purchased from Sigma Chemical Company (Steinheim, Germany). V-pyrro/NO (catalogue code: sc-205538, molecular weight:157.17) was procured from Santa Cruz Biotechnology, Inc. (Dallas, TX, USA). Distilled water and physiological saline (sodium chloride in water at a concentration of 90 mg/10 mL) wereobtained from Zentiva Pharmaceutical Company (Bucharest, Romania). Rat BDNF ELISA kit (catalogue code: ab213899) and MDA kit (catalogue code: ab118970) were acquired from Abcam Company (Waltham, Boston, MA, USA) and Ransod (SD125), and Ransel (catalogue code: RS505) kits were acquired from RANDOX Company (Warsaw, Poland).

### 2.2. Animals

This research was conducted using white Wistar rats weighing between 200 and 250 g, sourced from the “Cantacuzino” National Military Medical Institute for Research and Development Baneasa, Bucharest, Romania. The experimental protocol adhered to the guidelines recommended by the “Grigore T. Popa” University Committee for Research and Ethical Issues for the handling and utilization of experimental animals in accordance with the ethical standards set by the European Community. The research methodology was conducted in accordance with both international and national standards (Law No. 206/27 May 2004) [41]. 

These rats were acclimatized by arriving a day before the experiments, and they were housed under standard laboratory conditions, maintaining a temperature of 21 ± 2 °C, humidity levels between 50 and 70%, and a 12 h day/night lighting cycle. To eliminate potential chronobiological influences, the tests were conducted between 8 a.m. and 12 p.m. each day.

The rats were provided with standardized solid food (pellets), and the quantity of food consumed by each rat was measured daily. Access to drinking water was unrestricted and facilitated by specialized devices. On the day of the experiment, the rats were deprived of both food and fluids.

Standard laboratory food and tap water were freely available, except during the time of the experiments. To ensure accurate dosing of test substances throughout the experiment, the groups of animals were initially categorized and weighed using a precision automatic balance (Sartorius AG, Cambridgeshire, UK), accurate to a tenth of a gram. This allowed adjustments to be made to the test substance doses based on any weight variations observed in the animals during the course of the experiment.

Before the experiment, rats were placed on a raised wire mesh under a clear plastic box and allowed to acclimate to the testing room for 2 h.

The experiments involved a total of 40 animals. For every separate test, we employed a set with four indistinguishable batches of animals, each comprising five white male Wistar rats. The animals were treated intraperitoneally according to the following protocol:

Group I (Control): saline solution, 0.3 mL/100 g of body weight;
Group II (Mg): 200 mg/kg of body weight of magnesium chloride;
Group III (MSD): 1 mg/kg of body weight of molsidomine;
Group IV (V-pyrro/NO): 5 mg/kg of body weightof V-pyrro/NO.

The substances were administered in a solution of 0.6 mL, with the same volume for each. The animals received a single dose of the tested substances, and there were no substance associations.

Magnesium chloride (Mg) served as a reference control substance, possessing established effects on both behavioral models in rodents [42,43]. In our study, the dose of 1 mg of molsidomine used was exceptionally low and purportedly free from cardiovascular side effects.

After administering the substances, the animals were given a 15 min rest period before engaging in behavioral tests. Biochemical analyses were specifically performed on animals that underwent the actimeter test, and blood collection occurred immediately after the experimental session concluded.

### 2.3. The Spontaneous Behavior Evaluation

The rats’ psychomotor abilities were assessed using the LE-8811 Infrared Actimeter (Panlab, Harvard, MA, USA) to examine both their overall motor behavior and the frequency of escape attempts [44].

This behavioral model also provides crucial insights into the stress-related manifestations and anxiolytic-like tendencies of the animal when it is placed in an unfamiliar environment [45]. The actimeter test was selected because it has previously identified signs of anxiety-like behavior, as evidenced by the increased number of escape attempts, and its short session time minimized animal stress [46].

During testing, the rats were positioned within the device’s cage, and each movement generated a signal due to variations in the inductance and capacitance of the apparatus’s resonance circuit. These signals were automatically converted into numerical values. Horizontal and vertical (with a resolution at the millimeter scale) or stereotyped activity (at frequencies reaching up to 100 Hz) was quantified as the total number of beam interruptions over a 2 min interval. The examination of the data collected during the experimental session also uncovered additional parameters, such as total distance traveled and overall motor activity. These values were electronically recorded and displayed in accordance with pre-programmed settings through an electronic data-processing system.

### 2.4. The Short-Term Memory Evaluation

To explore the potential impact of the NO donors on spatial cognition ability, this study examined their influence on rat memory performance using a Y-maze test (Panlab, Harvard, MA, USA). This experimental model was used to assess the animal’s ability to recall the arm it had previously explored and subsequently choose one of the other arms within the maze [47,48].

During an 8 min session, each rat was initially placed at the end of one arm and allowed unrestricted movement throughout the maze. The first 2 min served as a habituation period, while the subsequent 6 min were dedicated to recording arm alternations using photobeam breaks positioned at the midpoint of each arm. An arm entry was only counted when a detector in a different arm was activated, disregarding repeated activation of the same detector.

Spontaneous alternation was defined as choosing the least recently visited arm, with alternation being the consecutive entry into three different arms. The alternation percentage was obtained using the following formula [49]:

[*number of alternations*/(*total number of arm visited* − 2)] × 100.

Supplementarily, the preference index (%) was also calculated according to the following formula [50]:

*time spent in the novel arm*/120.

A video camera set over the device and connected to a computer in another room was used to allow us to evaluate the behavior without distressing the animals. Supplementary variables, including the duration to exit the initial arm, the time spent on the initial visit to an arm, the overall count of arms visited, occurrences of revisiting alternate arms, and instances of returning to the same arm, were also recorded. All recorded trials were subsequently analyzed by an observer unfamiliar with the treatment condition. These tasks were conducted between 8:00 a.m. and 1:00 p.m.

### 2.5. Laboratory Investigations

Blood tests were conducted to identify alterations in specific stress-related parameters, including cortisol levels in the bloodstream, serum concentrations of brain-derived neurotrophic factor (BDNF), and the activity of some enzymes involved in oxidative processes, such as superoxide dismutase (SOD), malondialdehyde (MDA), and glutathione peroxidase (GPx). These investigations were carried out under 1% isoflurane anesthesia, and a 0.3 mL blood sample was obtained from the lateral tail vein using vacutainers containing ethylenediaminetetraacetic acid (EDTA) as an anticoagulant.

Serum cortisol level was assessed utilizing the immunochemical method featuring electrochemiluminescence detection (ECLIA Analyzer MSLCM12, Covina, CA, USA) [51]. The quantification of BDNF concentrations in the blood was carried out through the ELISA procedure (Automatic ELISA Apparatus, Shandong, China) [52]. SOD activity was determined (with Ransod kit) by spectrophotometric observation (at 505 nm) of superoxide anion production (Shimadzu 1800 Spectrophotometer, Kyoto, Japan) [53]. Plasma MDA levels were determined through a fluorescence detection method employing a high-performance liquid chromatograph (Agilent 1100 HPLC Series system, Santa Clara, CA, USA) and using an MDA kit [54]. The evaluation of GPx activity was conducted spectrophotometrically Shimadzu 1800 Spectrophotometer, Kyoto, Japan) using the dithio-nitrobenzoic acid reagent technique and a specific Ransel kit [55].

### 2.6. Data Analysis

The data were shown as mean values with accompanying standard deviations, and statistical significance was assessed using the ANOVA test carried out within SPSS Statistics 21.0 for Windows. Supplementarily, the Neumann–Keuls test was employed as a post hoc analysis. Statistical significance was defined as *p*-values less than 0.05 when compared to those of the control group.

## 3. Results

### 3.1. The Spontaneous Behavior Evaluation

**Figure 1:**
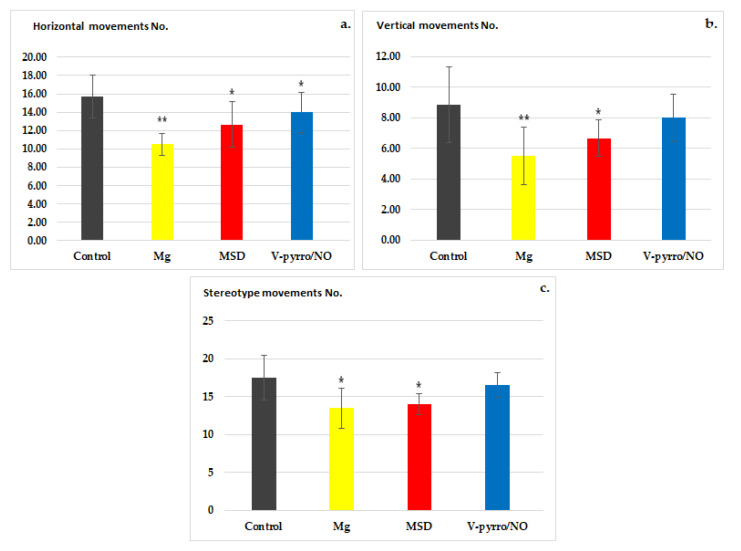
The influence of MSD and V-pyrro/NO on the horizontal (**a**), vertical (**b**), and stereotype (**c**) movements in the actimeter test. * *p* < 0.05 vs. control; ** *p* < 0.01 vs. control.

The actimeter test revealed that the use of Mg significantly reduced the locomotor activity of the rats compared to the control group. When assessing spontaneous behavior, Mg showed significant differences (** *p* < 0.01) in horizontal (10.50 ± 1.22 vs. 15.67 ± 2.34) and vertical (5.50 ± 1.87 vs. 8.83 ± 2.48) movements (Figure 1a,b) and a less-pronounced effect on stereotype (13.67 ± 2.66 vs. 17.33 ± 2.94) movements (* *p* < 0.05) compared to the group that received a saline solution (Figure 1c). Rats treated with MSD exhibited a notable decrease (* *p* < 0.05) in spontaneous activity in the horizontal (12.67 ± 2.50 vs. 15.67 ± 2.34) and vertical (6.67 ± 1.21 vs. 8.83 ± 2.48) planes, as well as in the number of stereotype (14.40 ± 1.38 vs. 17.33 ± 2.94) movements, in comparison to the control group (Figure 1a–c). The effects of MSD on the total locomotor activity were less pronounced than those of Mg in this experimental behavioral model in rats. V-pyrro/NO showed a significant variation (* *p* < 0.05) only in horizontal movements (14.00 ± 2.19 vs. 15.67 ± 2.34) (Figure 1a).

Both Mg and MSD significantly decreased the total distance covered by the animals in the actimeter test. Mg exhibited a more noticeable impact in reducing the distance traveled compared to MSD. The use of Y-pyrro/NO led to a decrease in the total distance covered, but this change did not reach statistical significance in comparison to the control group (Table 1).

During the experimental session, exposure to Mg and MSD caused a notable decline in overall motor activity compared to the saline group. While there was a decrease in global motor activity with V-pyrro/NO, this was not statistically relevant vs. the control (Table 1).

### 3.2. The Short-Term Memory Evaluation

**Figure 2:**
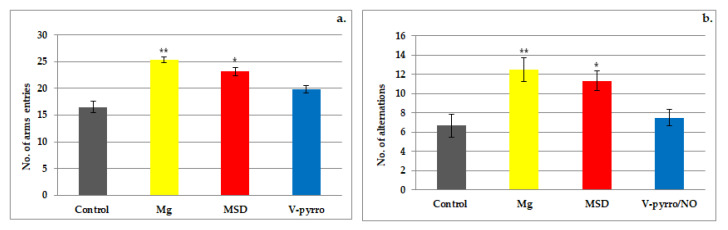
The influence of MSD and V-pyrro/NO on the arms visited (**a**) and number of alternations (**b**) in the Y-maze test. * *p* < 0.05 vs. control; ** *p* < 0.01 vs. control.

**Figure 3:**
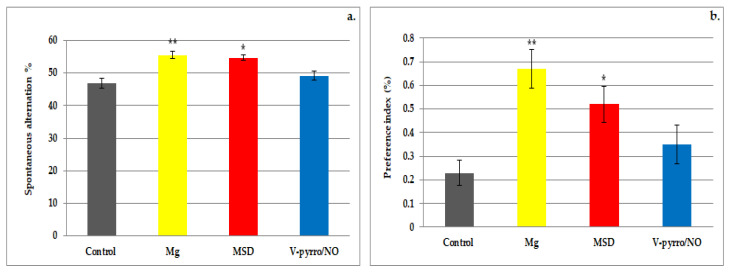
The influence of MSD and V-pyrro/NO on the spontaneous alternation percentage (**a**) and the preference index (**b**) in the Y-maze test. * *p* < 0.05 vs. control; ** *p* < 0.01 vs. control.

The administration of Mg led to a significant increase (** *p* < 0.01) in the number of arms visited (25.33 ± 0.52 vs. 16.50 ± 1.05) as well as in the number of alternations (12.50 ± 1.22 vs. 6.67 ± 1.21) when compared to the control group. In the assessment of short-term memory, it was observed that MSD treatment resulted in a more pronounced increase in activity compared to V-pyrro/NO treatment when compared to the group that received a saline solution (Figure 2a,b and Figure 3a,b). Rats treated with MSD showed a significant rise (* *p* < 0.05) in both the number of arms entered (23.17 ± 0.75 vs. 16.50 ± 1.05) and the number of alternations (11.33 ± 1.03 vs. 6.67 ± 1.21) compared to the control animals (Figure 2a,b). The effects of MSD were considerably less intense than those of Mg in this experimental model. In the Y-maze test, the use of V-pyrro/NO resulted in a slight increase but without any statistically significant changes in either the number of arms visited (19.83 ± 0.75 vs. 16.50 ± 1.05) or the number of alternations (7.50 ± 0.84 vs. 6.67 ± 1.21) compared to the control group (Figure 2a,b).

Both the use of Mg and MSD resulted in a significant increase in the rate of spontaneous alternation (55.5 ± 1.05 and 54.67 ± 0.82, respectively, compared to 46.83 ± 1.6) and in the preference index (0.67 ± 0.08 and 0.52 ± 0.08, respectively, compared to 0.23 ± 0.05) when compared to the control group. The effects were more pronounced for Mg (** *p* < 0.01) than for MSD (* *p* < 0.05) in this behavioral experimental model in rats (Figure 3a,b). The administration of V-pyrro/NO did not lead to any statistically significant changes in either the spontaneous alternation rate (49.14 ± 1.47 vs. 46.83 ± 1.6) or the preference index (0.35 ± 0.08 vs. 0.23 ± 0.05) when compared to the group that received a saline solution (Figure 3a,b).

### 3.3. Laboratory Investigations

**Figure 4:**
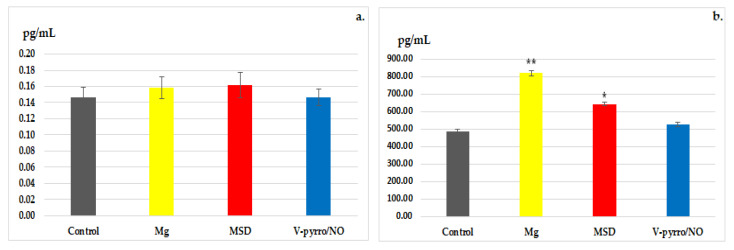
The influence of MSD and V-pyrro/NO on the blood levels of cortisol (**a**) and BDNF (**b**). * *p* < 0.05 vs. control; ** *p* < 0.01 vs. control.

In the biochemical estimation of reactivity to stress, no significant changes in blood cortisol levels were revealed for all three treated groups when compared to the control group (Figure 4a). However, when it comes to blood levels of BDNF, a statistically significant increase (* *p* < 0.05) compared to the control group (488.00±11.14) was observed in the MSD group (642.83 ± 11.55), and an even more pronounced increase (** *p* < 0.01) was noted in the Mg group (818.50 ± 13.37). The use of V-pyrro/NO did not result in substantial changes in serum BDNF values (526.50 ± 10.60) compared to the group that received a saline solution (Figure 4b).

**Figure 5:**
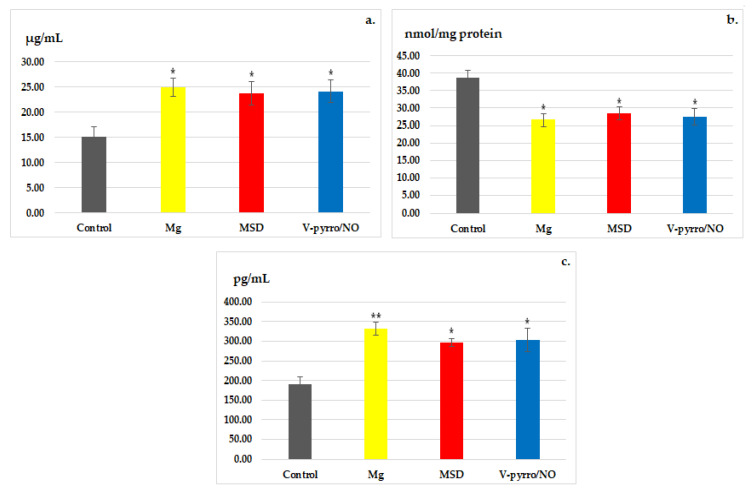
The influence of MSD and V-pyrro/NO on the activity of SOD (**a**), MDA (**b**), and GPx (**c**). * *p* < 0.05 vs. control; ** *p* < 0.01 vs. control.

In all three of the treated groups, a significant increase (* *p* < 0.05) was observed when compared to the control group with regard to the activity of SOD (Figure 5a). Conversely, there was a notable decrease (* *p* < 0.05) in the blood levels of MDA compared to the control group (Figure 5b).

Laboratory analysis revealed a significant increase in the activity of GPx as a result of Mg administration (** *p* < 0.01), and a less-pronounced increase (* *p* < 0.05) in GPx activity was observed for both MSD and V-pyrro/NO administration (Figure 5c).

## 4. Discussions

The comparative study of two distinct classes of NO donors (syndnones and diazeniumdiolates) originated from the observation that both demonstrate protective effects against chemically induced liver and kidney damage in rodents [38,56,57,58].

These investigations are components of a broader study focused on identifying the pharmacodynamic impacts of NO donors, particularly their influence on inflammation, oxidative stress, spontaneous behavior, exercise endurance, and cognitive functions in laboratory animals.

The selection of Mg as a reference treatment in our experiments is justified by the established connection between this trace element, crucial for body functions, and NO.

The literature indicates that Mg amplifies the generation of local vasodilators, such as prostacyclin and NO [59,60]. It boosts NO production, partly by increasing endothelial NO synthase levels [61]. Anomalies in Mg levels are implicated in cardiovascular disease pathogenesis, notably in conditions like hypertension, atherosclerosis, and thrombosis [62]. The revelation that low Mg concentrations exacerbate endothelial dysfunctions sheds light on the underlying mechanisms of these diseases [63]. Supplementation with Mg has been demonstrated to elevate NO synthesis, which plays a pivotal role in various functions such as vasodilation, ion channel regulation, and neurotransmitter release [64,65].

Endothelial cells within the brain produce NO endothelial NO synthases, chiefly impacting the baseline vascular tone while also regulating neuronal signaling and safeguarding the central nervous system against ischemic harm [66]. NO governs neurotransmission and neuronal metabolism and influences functions like learning, memory, and sleep [67].

A deficiency in Mg has been demonstrated to escalate the production of inflammatory mediators, triggering neuroinflammation that is believed to accelerate the progression of cognitive decline and dementia [68]. A recent meta-analysis of randomized controlled trials revealed that supplementation with Mg notably reduced serum C-reactive protein levels and boosted NO levels, a crucial vasodilator [69]. Within the brain, NO amplifies blood flow and plays a pivotal role in intracellular signaling among neurons [67].

The actimeter test is a straightforward sensorimotor assessment employed for the comprehensive evaluation of overall locomotor activity, stress-sensitive behavior, anxiety levels, and propensity for exploration in rodents [45]. The count of horizontal plane movements offers insights into the animal’s overall exploration of its environment, while stereotypical movements indicate self-maintenance behaviors related to personal hygiene (such as paw-licking, hair combing, nose cleaning, and other self-directed, potentially recuperative actions—autogrooming) [46]. The quantification of vertical plane movements provides insights into an animal’s efforts to ascend the see-through walls of the registration enclosure, serving as an indirect measure of escape attempt-based behavior, which in turn hints at the presence of fear and anxiety expressions [70,71].

Mg, as well as MSD, produced a notable inhibitory effect on locomotor activity and search behavior in the novel actimeter environment. Mg displayed a more pronounced effect on evaluated parameters than MSD. Although there was a slight decline in the general mobility after V-pyrro/NO compared with the saline group, it was not statistically significant in this behavioral model. Following the evaluation of the total locomotor activity, horizontal and vertical movements, as well as stereotyped movements, we observed that both Mg and MSD led to a reduction in spontaneous behavior in rats. This reduction included a decreased ability to explore their environment and fewer attempts to escape from unfamiliar surroundings. When we compared the results using the statistical analysis tools we employed, it became evident that Mg, which served as the positive control substance in the experiment, produced the most pronounced effects. On the other hand, treatment with V-pyrro/NO resulted in only a decrease in exploratory skills without significantly affecting other aspects of movement in our experimental conditions.

The Y-maze experimental model delves into spatial and recognition memory by capitalizing on rodents’ innate inclination to explore their surroundings.

In the Y-maze assay, an increase in the spontaneous alternation percentage is seen as an enhancement in spatial memory, particularly the short-term one, attributed to the administration of the substance tested. Conversely, a decline in the spontaneous alternation percentage indicates a disturbance in the animal’s short-term memory and spatial learning capacity [47]. The decrease in the animal’s entries into the arms of the apparatus is a consequence of the test substance’s ability to facilitate spatial memory. In contrast, an increase in arm explorations corresponds to an impairment in working memory [72].

Under our experimental conditions, the administration of Mg resulted in an enhancement of the rats’ short-term spatial memory, accompanied by a mild anxiolytic effect in the Y-maze test. The use of MSD was linked to improved recognition of previously explored arms, along with a slight anxiolytic effect. The impact of MSD on cognitive functions, particularly its ability to enhance spatial memory, was less pronounced than that of Mg in this experimental behavioral model. In contrast, treatment with V-pyrro/NO had no discernible effect on spatial memory and did not significantly influence the cognitive functions of the laboratory animals in the Y-maze test.

BDNF is found in various regions of the brain [73], and it plays a crucial role in maintaining and preserving neurons by regulating neurogenesis in specific brain areas. In recent years, different research groups have explored BDNF’s involvement in stress-related mental illnesses, particularly affective disorders, although the mechanisms responsible for these pathological states remain unclear. Stressors have been shown to impact brain function through two distinct systems: the stress-related system, primarily composed of the hypothalamic–pituitary–adrenocortical axis, the hippocampus, and the reward-related system, which mainly includes the ventral tegmental area and nucleus accumbens [74,75,76]. BDNF may exert specific influences on these two systems [77,78].

Affective disorders can lead to changes in the expression of neurotrophic factors like BDNF. The neurotrophic hypothesis of depression proposes that stress triggers a reduction in BDNF expression within crucial limbic structures, which are also implicated in the pathogenic mechanisms of depression [79]. This theory gains support from the observation that individuals with depression exhibit diminished levels of neurotrophins, known for their vital roles in shaping and adapting neural networks in specific brain regions. Additionally, BDNF expression is reduced, and its functions are altered in those with depression [80].

Blood tests conducted on the rats following the completion of the evaluation session in the actimeter test indicated significant increases in serum BDNF levels for Mg. However, there was no clear alteration in cortisol values.

The treatment with MSD resulted in a notable increase in BDNF blood levels, as well as in the activity of SOD and GPx. Additionally, there was a statistically significant decrease in MDA activity.

The impact of Mg was more pronounced than that of MSD. The substantial increase in the activity of the antioxidant enzymes, alongside the notable reduction in blood value of the pro-oxidant enzyme due to MSD and V-pyrro/NO, indicates the potential of these NO donors to diminish lipid peroxidation. This, in turn, suggests an enhancement in the rats’ oxidative status. The comparable intensity effects of the two NO donors on the parameters involved in tissue oxidation are inferior to those of Mg. These observed effects suggest the potential anxiolytic and antioxidant effects of MSD in rats.

An initial study demonstrated that MSD enhances experimentally induced performance deficits caused by 7-nitroindazole, a selective inhibitor of neuronal NO synthase that does not notably affect arterial pressure [81]. Subsequently, Pitsikas et al. conducted several studies examining the impact of MSD on rats experiencing memory impairment due to scopolamine treatment or the NO synthase inhibitor L-NAME. These investigations revealed a notable enhancement in cognitive disruptions within the object-recognition test, indicating that these effects might be mediated through the NOergic system rather than by enhancing cerebral circulation [82]. That research team showcased that MSD enhances behavioral changes and spatial recognition memory affected by the blockade of N-methyl-D-aspartate receptors using ketamine [33] or MK-801 in rats [83].

Unlike the outcomes observed by other researchers, our research findings, conducted using a small dose of MSD (1 mg/kg), revealed a clear enhancement in the natural cognitive abilities of rats. This improvement might be linked not only to the particular animal species and the limited sample size but also to the antioxidant properties displayed by this NO donor.

Additionally, the literature specifies that NO demonstrates varying effects based on dosage, with neuroprotective properties and beneficial impacts on cognitive functions noted with the administration of smaller doses of NO donors [84].

The neuroprotective attributes of low NO doses are proposed to stem from the biphasic dosing pattern inherent in the functionality of various neurotransmitters.

Certain authors suggest that the beneficial effects of minimal doses might arise from the interplay between the nitrinergic and glutamatergic systems [37]. They specifically note NO’s inhibition of NMDA receptors via S-nitrosylation. Lower doses could potentially counteract this inhibition, improving the activity of these glutamate receptors and thereby contributing to memory preservation [84].

It was observed that higher doses of MSD disrupted the rats’ learning ability as a result of the excessive production of NO and, implicitly, the accumulation of its harmful metabolite, peroxynitrite [85].

In the existing literature, conflicting findings exist regarding the effects of NO donors or precursors on anxiety-like behavior in animals. Some research suggests that administering NO precursors/donors such as sodium nitroprusside, L-arginine, NOC-9, or sildenafil leads to heightened anxiety. This conclusion was drawn from observations of animals spending reduced time in illuminated areas during light/dark tests and avoiding open arms in elevated plus maze tests [86,87,88,89,90]. Conversely, other studies indicate that NO donors may actually reduce anxiety in animals. This deduction is based on the increased time spent on open arms during elevated plus maze tests after injection and, in one study, a significant rise in the time spent in the central zone rather than the periphery during open field tests [37].

Several investigations indicated that the acute administration of the NO donors sodium nitroprusside or NOC-9 showed no changes in anxiety measures; overall locomotor activity; initial hesitation to enter the dark chamber; transitions between light and dark areas in the light/dark test; or the count of squares crossed, grooming instances, and upright standing in the open field test [91,92]. Similar results were seen with the NO precursor L-arginine in the light/dark assay [93].

However, in one study, outcomes varied based on dosage; a single acute dose of 1 mg/kg sodium nitroprusside led to behaviors suggesting reduced anxiety when administered 30 min before testing. In contrast, a single injection of 3 mg/kg decreased overall activity in rats when given 30 min prior to testing. Intriguingly, this same dose did not impact the animals’ performance in the light/dark or motor activity tests when administered 60 min before testing [94].

In a model of cognitive deficit induced by immunotoxin 192 IgG-saporin, Hernandez-Melesio et al. assessed the effect of MSD treatment on the spatial working memory of rats through an object-recognition test and showed that MSD avoided neuronal damage progression. The rats exposed to 192-IgG-SAP exhibited a reduction incortical nNOS expression vs. the control group, but expression was enhanced in the 192-IgG-SAP + MSD-treated animals [95].

Kalouda et al. evaluated the effects of MSD on anxiety-like behaviour and compared them with the anxiolytic diazepam in rats using the light/dark and the open field tests. MSD induced anxiolytic-like effects in the two tests, and the magnitude of the effect was not different to that produced by diazepam. Also, MSD did not alter locomotor activity compared with control rats in a motility test [37].

The results of our study are limited by both the reduced number of animals used during the experiment and the period of time for substance administration.

## 5. Conclusions

The use of MSD, but not of V-pyrro/NO, produced a diminution in exploratory and self-maintenance spontaneous behavior and anxiolytic effects in rats. Furthermore, the laboratory analyses conducted on the animals undergoing the actimeter test indicated that MSD administration resulted in a significant elevation of serum BDNF and exhibited notable antioxidant effects (the intensification of antioxidant enzymes, as well as SOD and GPx activity), albeit less potent than those observed with Mg.

We also proved that the treatment with MSD was associated with an improvement in rats’ cognitive abilities through the facilitation of short-time memory retention.

Additional research studies on a larger number of animals and a comparison of the effects to those of other NO donors should provide important perspectives for future clinical studies.

## Figures and Tables

**Table 1:** The influence of MSD and V-pyrro/NO on the total distance traveled and the global motor activity. * *p* < 0.05 vs. control; ** *p* < 0.01 vs. control.

	Total Distance Traveled (cm)	Global Motor Activity
Control	248.5 ± 42.35	165.4 ± 28.75
Mg	149.6 ± 18.52 **	107.7 ± 10.82 **
MSD	192.2 ± 34.67 *	122.5 ± 16.55 *
V-pyrro/NO	227.8 ± 39.33	149.3 ± 31.08

## Data Availability

Data, material, and software information are provided in the article.
